# A Direct MS-Based Approach to Profile Human Milk Secretory Immunoglobulin A (IgA1) Reveals Donor-Specific Clonal Repertoires With High Longitudinal Stability

**DOI:** 10.3389/fimmu.2021.789748

**Published:** 2021-12-06

**Authors:** Albert Bondt, Kelly A. Dingess, Max Hoek, Danique M. H. van Rijswijck, Albert J. R. Heck

**Affiliations:** ^1^ Biomolecular Mass Spectrometry and Proteomics, Bijvoet Center for Biomolecular Research and Utrecht Institute for Pharmaceutical Sciences, Utrecht University, Utrecht, Netherlands; ^2^ Netherlands Proteomics Center, Utrecht, Netherlands

**Keywords:** antigen binding fragment, secretory immunoglobulin A, mass spectrometry, human milk, clonal repertoire, antibody response

## Abstract

Recently, a mass spectrometry-based approach was introduced to directly assess the IgG1 immunoglobulin clonal repertoires in plasma. Here we expanded upon this approach by describing a mass spectrometry-based technique to assess specifically the clonal repertoire of another important class of immunoglobulin molecules, IgA1, and show it is efficiently and robustly applicable to either milk or plasma samples. Focusing on two individual healthy donors, whose milk was sampled longitudinally during the first 16 weeks of lactation, we demonstrate that the total repertoire of milk sIgA1 is dominated by only 50-500 clones, even though the human body theoretically can generate several orders of magnitude more clones. We show that in each donor the sIgA1 repertoire only changes marginally and quite gradually over the monitored 16-week period of lactation. Furthermore, the observed overlap in clonal repertoires between the two individual donors is close to non-existent. Mothers provide protection to their newborn infants directly by the transfer of antibodies *via* breastfeeding. The approach introduced here, can be used to visualize the clonal repertoire transferred from mother to infant and to detect changes in-time in that repertoire adapting to changes in maternal physiology.

## Introduction

A large part (~35%) of the proteins in our blood are immunoglobulins (Ig). These immunoglobulins, or antibodies, are used by our immune system to identify and neutralize pathogenic bacteria and viruses among others (1). A specific antibody recognizes a unique foreign epitope and can lead to an antigenic response resulting in either the recruitment of other (cellular) parts of our immune system or direct neutralization. Besides being present in blood, immunoglobulins are also present in other body fluids such as saliva and milk.

In human plasma (i.e., blood from which cells have been depleted) three dominant classes of immunoglobulins are present, namely IgG, IgA and IgM. Their concentration levels differ per individual but are generally highest for IgG 8.3-11.2 g/L, and somewhat lower for IgA 1.6-2.8 g/L and IgM 0.6-1.2 g/L (2). For human IgG, four sub-classes can be distinguished, IgG1, IgG2, IgG3 and IgG4, and for human IgA two sub-classes co-exist: IgA1 and IgA2.

In human milk the concentration levels of IgG, IgA and IgM are substantially lower, but also the order of abundance is different. In human milk, IgA is predominant by far at 1.0-2.6 g/L, with much lower abundances of IgG 9.6-20.4 mg/L and IgM 1.9-2.9 mg/L (3, 4). Moreover, the dominant IgA in milk is IgA1, and it appears largely as secretory IgA (sIgA), which is generally called a dimer, but is actually consisting of a J-chain connecting two or more IgA1 monomers bound to the secreted form of the polymeric immunoglobulin receptor (pIgR) protein, referred to as the secretory component (SC) (5). This complex will here be further called the IgA1 hetero-oligomer.

Very little is known on the origin of IgA clones in human milk. It has been shown in lab animal studies that B cells migrate from the gut-associated lymphoid tissue (GALT) to the mammary gland (6, 7). However, whether these B cells stay in the mammary tissue producing IgAs for longer periods of time is not known, given that a significant number of antibody producing cells are secreted in human milk (8).

Total plasma Ig levels are determined routinely in clinical practice because they provide key information on the humoral immune status. Low Ig levels define some humoral immunodeficiencies (9), whereas high levels are sometimes linked to liver diseases, chronic inflammatory diseases, hematological disorders, infections, and malignancies (10–12). Such immunofluorescence-based analyses primarily focus on total IgG or IgA levels and lack information about the concentrations of individual clones. Using dedicated assays, it is possible to focus on Ig sub-populations in the blood that bind a specific antigen, but these assays also target the likely polyclonal response against such a foreign element (13).

Recently, we introduced a novel LC-MS based approach to directly monitor the levels of IgG1 in plasma, whereby we could not only monitor the total plasma levels of IgG1 in donors, but were also able to distinguish and quantify the abundance of the 50-500 most abundant individual IgG1 clones present in plasma (14). Strikingly, we observed that the IgG1 repertoires were unique for each donor and remained quite stable over time in each healthy individual donor when monitored longitudinally. In that work we used affinity beads to capture all IgG molecules from plasma, and subsequently used the enzyme IgdE which cleaves off the Fab parts solely of IgG1 molecules. By collecting these Fab molecules and analyzing them by intact LC-MS we were able to distinguish and quantify distinct IgG1 clones. We demonstrated that this method can be used to monitor the changes in abundance of individual clones in the plasma IgG1 repertoire over time, for instance caused by an infection (14).

Here we describe how we extended this method to target another important class of immunoglobulins, namely IgA1. Because of the different nature of these IgA1 molecules, we needed to adopt the reported approach for IgG1 substantially. We again used affinity resins, albeit now to capture specifically IgA molecules from human milk, and subsequently used the *O*-glycopeptidase from *Akkermansia muciniphila* (OgpA, commercially available as OpeRATOR^®^) that cleaves IgA1 molecules in their *O*-glycosylated hinge region, releasing the Fab fragments. These Fab fragments, of around 48 kDa, are subsequently analyzed by intact protein LC-MS enabling us to distinguish and quantify distinct IgA1 clones. The method was first optimized using a recombinant monomeric IgA1 (mIgA1), and subsequently we show that the method works equally well on repertoires of sIgA1 from human milk and IgA1 repertoires purified from plasma.

Having established the qualitative and quantitative reproducibility of the method, we next used it to longitudinally monitor the IgA1 repertoires in human milk of two individual donors over a period of several weeks. We observed that the sIgA1 clonal repertoires in human milk are dominated by a limited number of clones. Furthermore, monitoring the longitudinal samples from two healthy donors allowed us to reveal that the donors are both unique in their human milk sIgA1 clonal repertoire. Within a single healthy donor these IgA1 profiles are highly stable over time.

The method introduced here will be useful to monitor the humoral immune status both in human milk and plasma. Additionally, it may provide information on the personalized response towards an infection and/or therapeutic treatment through changes in the IgA1 clones being presented in plasma and human milk, and likely in other biofluids as well. As sIgA1 immunoglobulins are the main antibodies transferred to the newborns *via* breastfeeding, we feel that monitoring these sIgA1 repertoires may furthermore be used to monitor the immune status of the donor’s milk.

## Materials and Methods

### Human Subjects and Samples

**Figure 1:**
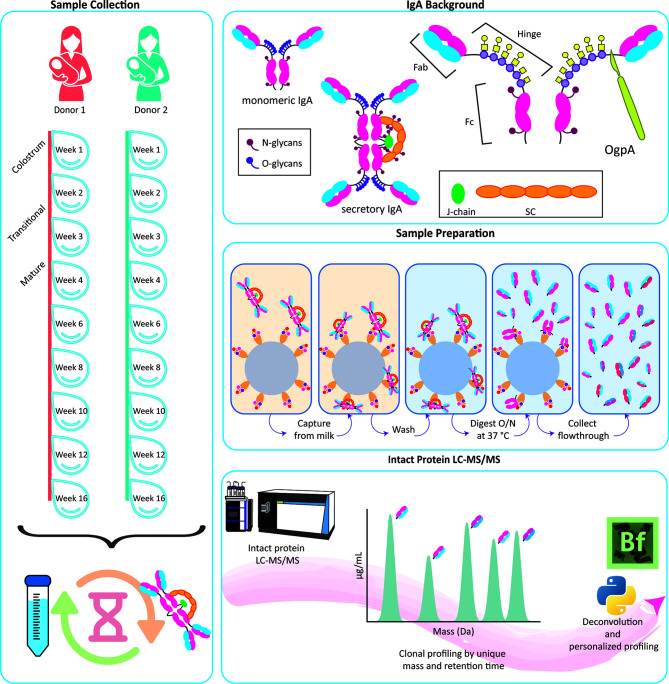
Personalized human milk sIgA1 Fab clonal profiling. Longitudinal human milk samples were obtained, with consent, from two healthy donors across weeks 1-16 of lactation (left panel). Following efficient capture of sIgA1, using CaptureSelect IgA affinity matrix beads, we relied on the *O-*glycopeptidase from *Akkermansia muciniphila* (OgpA), cleaving N-terminally of the *O*-glycans that are exclusively present in the IgA1’s hinge region (top panel), to dissect and collect their Fab fragments. The protocol works equally well, for capture and digestion, for human milk sIgA1 and plasma-derived or recombinant monomeric IgA1 (middle panel). The eluted Fab molecules were subsequently mass analyzed by reversed phase LC-MS, and masses were retrieved from the generated RAW files using BioPharmaFinder with additional data analysis performed using Python (bottom panel).

Details of the two donors have been described before (15). Longitudinal human milk samples were collected from two healthy donors at weeks 1, 2, 3, 4, 6, 8, 10, 12 and 16 postpartum (**Figure 1**). Samples were collected according to standardized human milk handling conditions (16). All samples were collected into 2 mL Eppendorf tubes containing protease and phosphatase inhibitors as 1/9 of the collection volume, Complete Mini EDTA-free (Roche) and PhosSTOP (Roche), respectively. Samples were transferred to the lab on dry ice and stored at -80°C until analysis. Written informed consent was obtained prior to sample collection. All samples used were donated to Danone Nutricia Research in accordance with the Helsinki Declaration II.

Monoclonal anti-CD20 mIgA1 (7D8-IgA1) was a gift from Genmab (Utrecht, NL). SIgA from human colostrum was obtained from Merck (Darmstadt, Germany).

Normal human recovered plasma was purchased from ZenBio (Durham, NC, US).

### IgA Enrichment

All IgA was captured using CaptureSelect IgA affinity matrix (ThermoFisher Scientific). 40 µL bead slurry was added directly to Pierce spin columns with screw cap (ThermoFisher Scientific). The beads were then repeatedly washed with 150 µL PBS by centrifugation at 500 × *g*, room temperature (RT). After the third wash, a plug was inserted to the bottom of the individual spin columns and 100 µL PBS was added to the beads. Subsequently 50 µL of human milk and 1 µL IgA solution containing 200 ng of 7D8-mIgA1 were added. Samples were then incubated for 1h while shaking at 750 rpm at RT in and Eppendorf thermal shaker (Eppendorf, The Netherlands). Following the incubation, the plugs were removed from the spin columns, the human milk/PBS dilution was collected by centrifugation for 1 min at 500 × *g*, RT. Then the beads were washed four times by addition of 200 µL PBS and subsequent centrifugation for 1 min at 500 × *g*, RT. After the fourth wash the plugs were reinserted into the bottom of the spin columns.

### IgA1 Hinge Region Digestion and Collection of Fabs

For the hinge region digestion of IgA1 we relied on the *O*-glycopeptidase from Akkermansia muciniphila, OgpA; OpeRATOR^®^, Genovis, Llund, Sweden). The enzyme docks at *O*-glycans, by preference non-sialylated core 1 (thus GalNAc-Gal), and then cleaves the protein N-terminally of the glycan. As these *O*-glycans are unique for IgA1 (and not present in IgA2) exclusively the Fab molecules of IgA1 are cleaved off.

Based on previous analysis of these human milk samples, by MS-based proteomics and ELISA, the expected IgA1 concentrations in the human milk samples were assumed to be 0.2 - 1.4 mg/mL (17), and therefore contain at least 10 µg in 50 µL. Based on these concentrations we added to each spin column 50 µL PBS containing 40 U SialEXO (a sialidase cocktail to remove sialic acids from the *O*-glycans) and incubated for 1 h at 37°C with continuous shaking at 750 rpm. Then 1 µL (40 U) of OgpA enzyme was added, and incubation was continued overnight, in and Eppendorf thermal shaker (Eppendorf, The Netherlands). Following overnight digestion with OgpA, 20 µL of pre-washed Ni-NTA agarose slurry (1:1 in PBS) was added to the spin columns in order to capture the His-tagged enzymes. The incubation was continued for 30 more minutes. Then the plug was removed from the column, and the flowthrough, containing the IgA1 Fabs, was collected by centrifugation for 1 min at 500 × *g*, RT.

### SDS-PAGE Gel Electrophoresis

The capturing and digesting efficiency of IgA1 Fabs were assessed by SDS-PAGE gel. Two standards were used, a commercially available human colostrum sIgA sample (Merck, Darmstadt Germany) and the recombinant monoclonal 7D8-mIgA1. They were captured and digested as stated above. The human colostrum sIgA and 7D8-mIgA1 were made as concentrations of 40 µg of protein in a 1% milk powder background (Bio-Rad, The Netherlands) in PBS. Starting material from both the human colostrum sIgA and 7D8-mIgA1 were collected, as were the flow through from capturing, the capture eluate, Fab and Fc. The samples were transferred onto the gel as 8 µg of protein per sample and diluted with the XT sample buffer to reach the desired concentration (Bio-Rad, The Netherlands). All samples were analyzed under non-reducing conditions. A volume of 45 µL of each sample was loaded onto a 12-well 4-12% CiterionTX MT Bis-Tris Prease Gel (Bio-Rad, The Netherlands). Precision Plus Protein Dual Color Standards (Bio-Rad, The Netherlands) were ran on the gel in parallel with the samples for protein size reference. The chamber was filled with 500 mL of XT MOPS running buffer (Bio-Rad, The Netherlands) pre-diluted 20 times with MilliQ water (Merck Millipore, Germany). Electrophoresis was carried out for 2 h and 30 min at 200 V until the dye front ran down to the bottom of the gel. The gel was then washed with MilliQ three times followed by staining for 1 h in Imperial Protein Stain (Thermo Scientific, Rockford, IL USA). The stained gel was then rinsed overnight in MilliQ followed by scanning using an Amersham Imager 600 (GE Healthcare, USA).

### Fab Profiling by LC-MS

The same LC-MS and data processing approaches as described by Bondt et al. were applied (14). In short, the collected Fab samples were separated on a Thermo Scientific Vanquish Flex UHPLC instrument, equipped with a 1 mm x 150 mm MAbPac Reversed Phase HPLC Column. The LC was directly coupled to an Orbitrap Fusion Lumos Tribrid mass spectrometer (Thermo Fisher Scientific, San Jose, California, USA). The column preheater and the analytical column chamber were heated to 80°C during chromatographic separation. 10 µL of the prepared Fab samples were injected and subsequently separated over a 62 min gradient at a flow rate of 150 µL/min. Gradient elution was achieved by using two mobile phases A (0.1% HCOOH in Milli-Q HOH) and B (0.1% HCOOH in CH_3_CN) at a starting mixture of 90% A and 10% B, and ramping up from 10% to 25% B over 1 min, from 25% to 40% over 54 min, and from 40% to 95% over 1 min. MS data were collected with the instrument operating in Intact Protein and Low Pressure mode. Spray voltage was set at 3.5 kV from minute 2 to minute 50 to prevent the salts in the sample from entering the MS, ion transfer tube temperature was set at 350°C, vaporizer temperature at 100°C, sheath gas flow at 15, auxiliary gas flow at 5, and source-induced dissociation (SID) was set at 15 V. Spectra were recorded with a resolution setting of 7,500 (@ 200 *m/z*) in MS1, recording at low resolution allows for better detection of charge distributions of large proteins (> 30 kDa) (18). Scans were acquired in the range of 500-4,000 *m/z* with an AGC target of 250% and a maximum injection time set to 50 ms. For each scan 5 µscans were recorded.

### IgA1 Clonal Profiling Data Analysis

Intact masses were retrieved from the generated RAW files using BioPharmaFinder 3.2 (Thermo Scientific). Deconvolution was performed using the ReSpect algorithm between 5 and 57 min using 0.1 min sliding windows with a 25% offset and a merge tolerance of 50 ppm, and noise rejection set at 95%. The output mass range was set from 10,000 to 100,000 with a target mass of 48,000 and mass tolerance 30 ppm. Charge states between 10 and 60 were included, and the Intact Protein peak model was selected.

Further data analysis was performed using Python 3.8.10 (with libraries: Pandas 1.2.3 (19), Numpy 1.20.3 (20), Scipy 1.6.2 (21), Matplotlib 3.3.4 (22) and Seaborn 0.11.1). Masses of the BioPharmaFinder identifications (components) were recalculated using an intensity weighted mean considering only the most intense peaks comprising 90% of the total intensity. Using the 7D8-mIgA1 standard, the intensity was normalized, a relative mass shift was applied to minimize the mass error and a retention time shift was applied to minimize deviation between runs.

Components between 45,000 and 53,000 kDa with the most intense charge state above *m/z* 1000 and score of at least 40 were considered Fab portions of IgA1 clones. The clones were matched between runs using average linkage (unweighted pair group method with arithmetic mean UPGMA) L^∞^ distance hierarchical clustering. Flat clusters were formed based on a cophenetic distance constraint derived from a mass and retention time tolerance which were 2 Da and 1 min respectively. Clones within a flat cluster were considered identical between runs. To determine the range of quantification of IgA1 Fab clonal profiling, the human colostrum sIgA and 7D8-mIgA1 standards were made in various concentration ranges, captured, digested, and analyzed by MS in triplicate. See **Supplemental Table S2** for details.

## Results

### Optimization of the Protocol for Enrichment and Fab Cleavage of a Recombinant Monomeric IgA1 and Milk sIgA1

**Figure 2:**
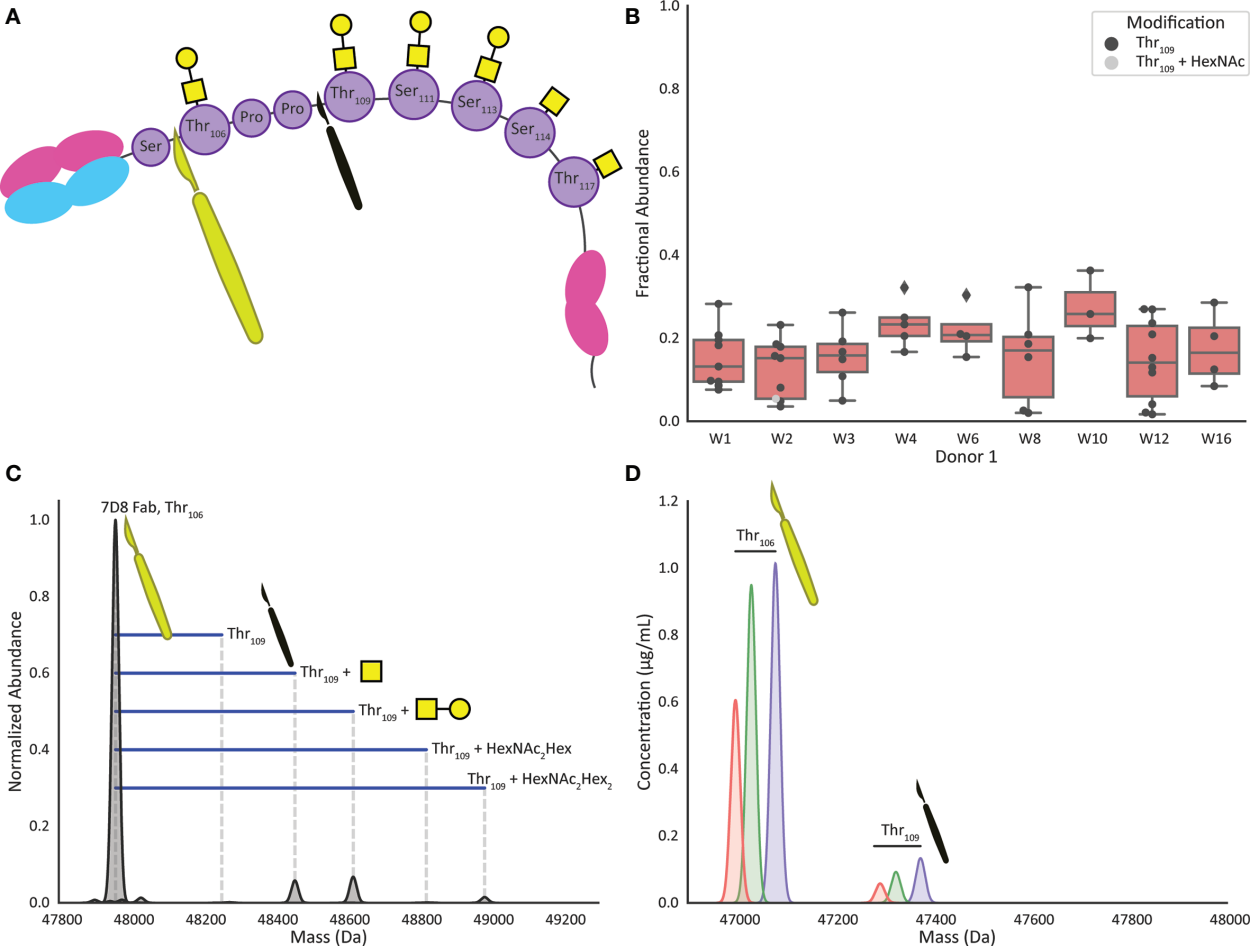
Digestion by OgpA of recombinant mIgA1 and human milk sIgA1 results in highly specific cleavage and formation of Fab fragments. **(A)** Cartoon of an IgA1 molecule highlighting the preferred cleavage site of *O*-glycopeptidase from *Akkermansia muciniphila* (OgpA). The large yellow scalpel indicates the preferred Thr_106_ site, the smaller black scalpel shows the observed missed cleavage site (digestion at Thr_109_) adding Thr_106_Pro_107_Pro_108_ to the Fab sequence. **(B)** The boxplots show the fractional abundance of the Fab fragments resulting from the Thr_109_ missed cleavage as compared to the corresponding Thr_106_ base peak. When observed, the Thr_109_ missed cleavage is generally lower than 20% in abundance compared to the Thr_106_ peak, and almost exclusively non-glycosylated at Thr_106_. **(C)** Overnight incubation of monoclonal mIgA1 with OgpA resulted in highly selective digestion at the *O*-glycosylation site Thr_106_, as determined by the detection of the calculated mass of the Fab. Minor satellite signals were detected, originating from the missed cleavage at Thr_109_, carrying one HexNAc, HexNAc+Hex, or 2(HexNAc+Hex). **(D)** Extracted ion chromatograms of three selected Fab clones in Donor 1 week 1 that display highly selective digestion at *O*-glycosylation site Thr_106_, only one additional signal can be observed, digestion at Thr_109_ carrying no *O*-glycan at Thr_106_ position.

Human IgA1 (UniProt IGHA1_HUMAN, P01876) contains up to six *O-*glycans in the hinge region (**Figure 1**). Supposedly three of these are always occupied, residing on Threonine (Thr)_106_, Thr_109_ and Serine (Ser)_113_, whereas Ser_111_, Thr_114_ and Thr_117_ are not always *O*-glycosylated (23, 24). Considering the specificity of OgpA, which cleaves exclusively N-terminally of (core 1) *O*-glycosylated residues, our rationale was that, when incubating for sufficient time, all heterogeneity introduced by the *O-*glycans could be removed and all IgA1 Fabs would share the same C-terminus due to cleavage N-terminal of Thr_106_ (**Figure 2A**).

#### MS-Based Assessment of the OgpA Cleavage Specificity

First, we used a recombinant monomeric anti-CD20 IgA1 antibody (7D8; a gift from Genmab, Utrecht, NL). The theoretical average mass of this Fab, 47957.4 Da, could be calculated based on its known sequence (**Supplemental Table S1** **)**. Following the approach as depicted in **Figure 1** we enriched this IgA1 antibody and subjected it to overnight cleavage by OgpA. Subsequently, we analyzed the released Fab fragments by LC-MS. Mass analysis indeed indicated that the main observed Fab fragment exhibited the expected mass when cleaved at N-terminal of Thr_106_ (**Figure 2A**). Additionally, lower abundant masses could be assigned to a ‘missed cleavage’ at Thr_109_. The observed mass increment was +295.3 Da (in agreement with the mass of the additional ThrProPro amino acid sequence (**Figure 2C**), decorated with either one N-acetylhexosamine (HexNAc, +203.2 Da), one HexNAc and one hexose (Hex, +162.1; HexNAcHex = +365.3) or a mass that fits with a potential core 2 *O-*glycan consisting of two HexNAcs and two hexoses (+730.7 Da). These masses were detected at 5.8%, 6.8%, and 1.5% intensity relative to the dominantly abundant base-peak Fab species, respectively.

Next, we applied the same approach on sIgA from human milk and found that both capturing, and digestion were equally successful for sIgA1. Of note, IgA2 will also be captured by the affinity resin, but not cleaved by OgpA as it does not contain any *O*-glycans in the hinge region. **Figure 2D** shows the extracted ion chromatograms of 3 illustrative Fab clones detected for sIgA, enriched from a human colostrum sample (donor 1 week 1). Also, for these three sIgA Fab clones, the cleavage is observed mainly at Thr_106_. Considering all clones present at a concentration > 0.5 µg/mL roughly 10-30% of the clones showed the additional Thr_109_ ‘missed-cleavage’, typically with an intensity below one-fifth of the main peak (**Figure 2B**). Unlike the monoclonal mIgA1, no Thr_109_ cleavage variants carrying *O-*glycans on Thr_106_ were observed in the human milk sIgA1 Fabs.

#### Gel-Based Assessment of the OgpA Cleavage Efficiency

To further assess the capturing and digestion efficiency of the approach, we further tested our procedure by using a commercial human colostrum sIgA standard (containing both sIgA1 and sIgA2) and a recombinant mIgA1. Both standards were prepared at equivalent concentrations. For both the sIgA and mIgA1 standards, a milk-based blocking buffer was added to reduce sample loss during the preparation. The starting material, flowthrough (FT) and eluate of the capture, and flowthrough and eluate after digestion were analyzed by SDS-PAGE gel electrophoresis. In **Supplemental Figure S1** we see that the starting material (lanes A1 and B1) appeared as expected at >250 kDa for the sIgA, and at ~160 kDa for the mIgA1. For both the sIgA and mIgA1 the flowthrough after capture were found to be devoid of IgA (lanes A2, A4, B2, and B4), indicating that all initial IgA was efficiently captured by the beads regardless of the IgA oligomeric state or sub-class. The eluate from the beads (lanes A3 and B3) contained the same IgA bands as the starting material, and strongly reduced background bands. The digested standards showed Fab bands in the flowthrough at ~50 kDa for both samples (lanes A5 and B5). The sIgA digestion eluate (lane A6) showed a band that was shifted to a lower mass due to the loss of four Fab portions, and a smear at higher molecular weight which is assumed to be sIgA2, as it would have been captured but not digested by OgpA. The band in lane B6 at ~160 kDa appears to be slight under-digestion, whereas the area at around ~60 kDa contains the highly glycosylated Fc. Altogether, this shows that our approach is able to capture and digest with equivalent efficiency recombinant mIgA1 as well as polyclonal hetero-oligomeric sIgA1.

### Quantification of Human Milk sIgA1 Clonal Repertoires

**Figure 3:**
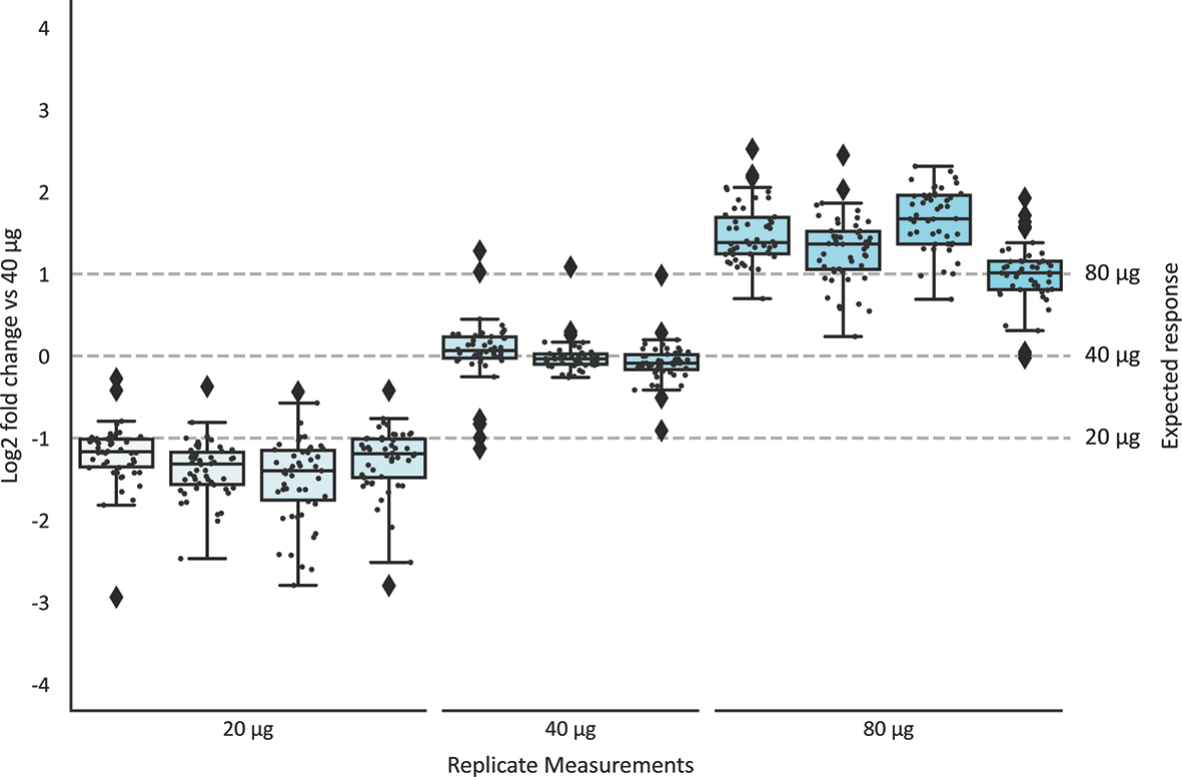
Accuracy and reproducibility of quantification of individual sIgA clones. The top 50 most intense clones from a human milk colostrum sIgA standard were quantified relative to a monoclonal mIgA1 of known quantity. The detected quantities of each clone in all samples were compared to the levels in one sample with 40 µg sIgA, and the fold-change was plotted. Replicates of the same injection amount (40 µg) showed no significant fold-change. Clone intensities in the 20 µg replicates are detected at half the abundance (-1 log_2_ fold change), and clone intensity in the 80 µg replicates were detected at double the abundance. Boxplots indicate the median, 25^th^ and 75^th^ percentile, whiskers range to 1.5 times IQR. Values inside this range are depicted as black dots. Outliers (> 1.5 times IQR) are indicated as black diamonds. **Supplemental Figure S2** depicts similar boxplots, considering all clones detected in all replicates (n=128) instead of the top50 most intense clones shown here.

To determine the concentration of individual sIgA1 clones in human milk, a known quantity of the monoclonal mIgA1 was spiked-in at the start of the sample preparation. Since we demonstrated above that the mIgA1 undergoes a similar capturing, digestion, and MS-analysis, it is possible to normalize the intensity of the individual sIgA1 clones to the measured intensity of the recombinant mIgA1 clone, and subsequently convert the MS intensity values to a concentration in µg/mL. To test the accuracy and reproducibility of this quantification, the standard quantity of the mIgA1 was added to different quantities of the human colostrum sIgA standard, see **Supplemental Table S2**. For the most intense clones detected in the human colostrum sIgA standard, the intensity values of each replicate were compared to one of the 40 µg sIgA standards, and the intensity fold-change of each individual clone was determined (**Figure 3**; **Supplemental Figure S2**). Replicates that also contained 40 µg sIgA show on average no change in the detected clone intensity, while clones of 20 µg replicates are detected at half the intensity (-1 log_2_ fold change) and clones of 80 µg replicates are detected at double the intensity (+1 log_2_ fold change). Within replicates of the same amount of sIgA, clones are reliably detected at similar intensity values. From these data we conclude that we can reproducibly detect the same clones across multiple technical replicates, and that the spiked-in mIgA1 allows for robust and reproducible quantification of individual sIgA1 clones. Of note, the quantification of low abundant clones from 20 µg sIgA was less robust than for 40 µg (**Supplemental Figure S3**).

### The Human Milk sIgA1 Repertoire Is Dominated by a Limited Number of Stable Clones, Being Unique for Each Healthy Donor

Having established that the capturing, digestion, and quantitation of individual clones of sIgA1 could be performed efficiently and reproducibly, we next applied the protocol to human milk obtained, with consent, from two healthy individuals. Samples were collected longitudinally over a span of 16 weeks, in total nine different time points per individual. We investigated these human milk samples to gain insight into a) the potential sIgA1 clonal diversity between individuals and b) the potential (dis)similarity in the sIgA1 repertoires within an individual over time. Using the approach described above we were able to detect for donor 1 a median of 537 (range 437 to 653) different pairs of mass and retention time that fitted the criteria to originate from distinct sIgA1 Fab clones, and for donor 2 we were able to detect around 460 clones per time point. Stringent analysis revealed that about 7-10% of these distinct ion signals matched to a Thr_109_ ‘missed cleavage’, thus leaving still ~400 unique clones. Thus, as earlier observed for IgG1 in plasma (14), we found that in human milk a limited number of clones dominate the sIgA1 repertoire.

**Figure 4:**
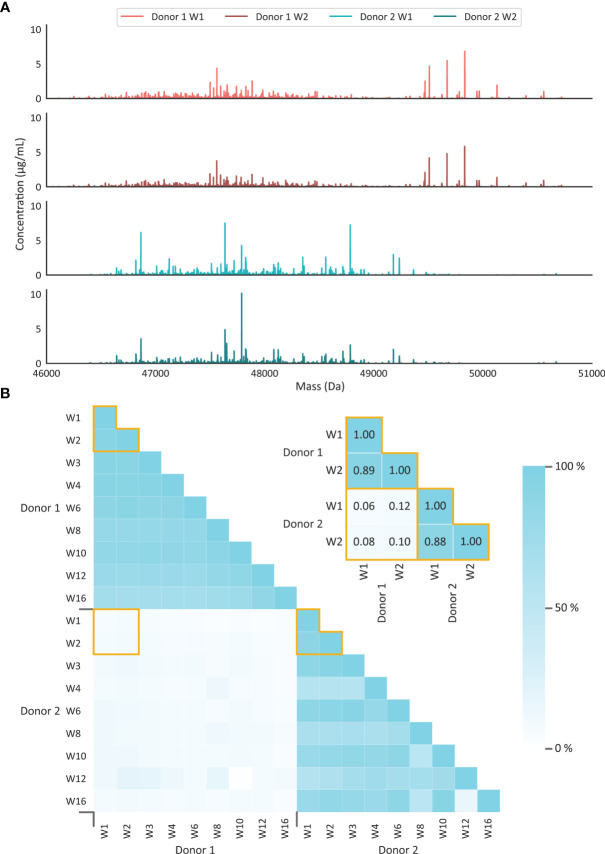
Human milk sIgA1 clonal profiles are stable over time, and highly unique for every donor. **(A)** Illustrative deconvoluted Fab mass profiles with the top two originating from donor 1 with milk obtained at weeks 1 and 2, and the bottom two originating from donor 2 with milk obtained at weeks 1 and 2. Each peak represents a unique Fab (based on unique RT/mass pair) and the peak height represents the concentration of that clone in the milk. The sIgA1 profiles obtained at all other time points are provided in **Supplemental Figure S5**. Clearly, just a limited number of distinct clones dominate the sIgA1 repertoire in each of the two donors. **(B)** Observed overlap in clonal sIgA1 repertoires within and in between donors. The persistence of repertoires is given as a percentage of the total sIgA1 clone abundance. Each small square depicts a percentage, as indicated by the color bar, of overlap between the samples. The inset depicts a zoom-in of the data for the profiles shown in **(A)**, annotated with the overlap values. The overlap in the sIgA1 repertoire is ~80% when comparing samples of the same donor, even when samples were collected as much as 16 weeks apart. Between donors hardly any overlap in the repertoires is observed (below ~10%), whereby even each clone seems to be uniquely detected in just one of the donors.

Comparison of the sIgA1 Fab clonal profiles, obtained from all our eighteen analyzed milk samples, showed a very high degree of overlap between the different time points when solely focusing on one, or the other, individual donor (**Figure 4** and **Supplemental Figure S4**). Although a gradual decrease in overlap was observed over time, even at 16 weeks still 80% of the total detected sIgA1 Fab molecules originated from clones already detected at week 1. From this we conclude that the sIgA1 Fab repertoires of individual donors are relatively simple, dominated by just a few hundred sIgA1 clones, and that in healthy donors this repertoire changes only marginally over the first 16 weeks of lactation. In contrast, when comparing the repertoires of the individual donors with each other, we detect no overlap. From this we conclude that, just as observed earlier for serum IgG1 repertoires (14), the clonal repertoires of human milk sIgA1 are highly unique for each individual.

### The Majority of sIgA1 Clones Show a Gradual Similar Decreasing Trend From Colostrum to Mature Milk

**Figure 5:**
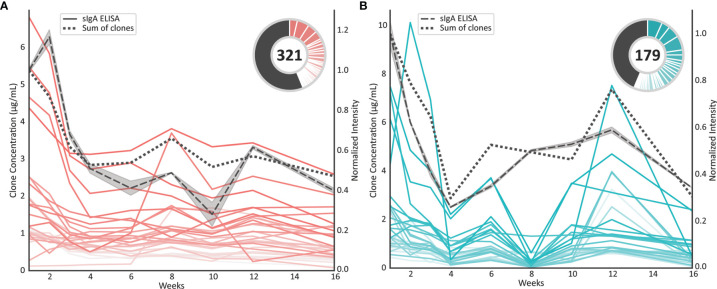
Concentration profiles of individual sIgA1 clones compared to the total sIgA concentration profile. **(A)** Longitudinal individual sIgA1 concentration profiles observed for donor 1. Each pink line represents a unique sIgA1 clone. The dotted black line represents the summed concentrations over all detected sIgA1 clones, whereas the dashed black line represents the total sIgA concentration as evaluated by an independent sandwich ELISA. The relative abundance of the top 30 clones at week 1 are shown in the pie charts, where the number in the center indicates the total number of clones that could be monitored longitudinally. For these analyses clones were included when present in at least 7 of the 9 time points. The top 30 clones each have a slice in the pie chart and the remainder of the clones are depicted as black pie slice. **(B)** As in A) individual sIgA1 concentration profiles observed for donor 2. Each blue line represents a unique sIgA1 clone.

Colostrum is the milk that is produced within the first few days postpartum. It is known to have a much higher concentration of sIgA compared to mature milk, whereby this concentration gradually declines throughout the first six months of lactation (25). For the sample set studied in the current manuscript we have reported the total sIgA levels determined by ELISA (17). Now, we compared the readout from this ELISA to the MS-based quantification of the profiling method described here (**Figure 5**). When comparing the total sIgA ELISA data (grey dashed line) to the sum of concentrations of all individual sIgA1 clones detected here (black dotted line), the longitudinal profiles are highly correlated (Pearson correlation coefficient r_donor1_ = 0.89, r_donor2_ = 0.89), for each of the two donors. The uniqueness of the present method is that individual clones can be monitored longitudinally, and thus the longitudinal profiles of the top 30 most intense clones in each of the donors are depicted as pink and blue lines in **Figure 5** as well. Additionally, the contribution of these clones to the total repertoire is shown in the pie chart. The total number of clones is given inside the pie chart and the concentration of the remainder of clones that are lower in abundance than the top 30 clones are depicted as the black pie slices. Monitoring the longitudinal clonal behavior showed that the majority of sIgA1 clones decreased in concentration proportionally to the overall decrease in total sIgA. Nonetheless, also some clones could be observed that revealed a longitudinal pattern deviating from the total sIgA.

### Application of the Protocol on a Plasma IgA1 Repertoire

Although here optimized and used for analysis sIgA1 repertoires in human milk, we wanted to further explore the general usability of the approach introduced here and applied, as proof-of-concept, the method on a plasma sample from a single healthy donor. We reiterate, as described in the introduction, distinctively from milk, in plasma most IgA1 is thought to be present in a monomeric form (5). **Supplemental Figure S6** shows the obtained IgA1 mass profile for this sample. The pie inset shows that 184 unique Fab clones could be detected. The top 30 most abundant clones were colored in both the mass profile and the pie inset, and these top 30 most abundant clones contribute roughly 50% of the total abundance of identified Fab molecules in this plasma sample. This shows, as a proof-of-concept, that our approach works equally well for plasma, and that the IgA1 repertoire from plasma is also dominated by a limited number of clones, just as the IgG1 repertoire (14).

## Discussion

Here we introduce a fast and sensitive approach for analyzing and monitoring IgA1 clonal repertoires from complex biofluids, such as human milk and plasma, revealing that the method is unbiased towards mIgA1s or hetero-oligomeric sIgA1s. Applying our approach to analyze human milk samples from two individual donors longitudinally over a 16-week period of lactation, we found that the human milk sIgA1 clonal repertoires are dominated by a limited number of around 50 - 500 stable and unique clones. This observation is quite striking as in theory the human body can make millions or more different antibodies. However, this observation is fully in line with our recent reporting on the IgG1 repertoires in human plasma, which were also found to be dominated by 50 - 500 stable and unique clones (14). In part, it can be explained by the limited number of B cells present locally in the mammary glands, although this number likely still exceeds the number of detected clones by several orders of magnitude.

So far, the accessibility of individual Igs clones has been deemed too difficult due to their complex structural aspects, heterogeneity, and immense sequence variability, which hampers most analytical approaches. A rather successful alternative, that is regularly applied, involves sequencing the Ig-genes from B cells (26–30). This approach indeed provides antibody sequences, but these are likely not identical to the fully matured protein sequence of the Igs that are finally produced and secreted (31). We largely overcome these limitations by analyzing the Ig profiles of human biofluids directly, which we demonstrate here for sIgA1 from human milk and plasma, and have previously shown for IgG1 from human plasma (14).

In this approach each clone is defined by having a unique set of identifiers, namely its retention time in liquid chromatography and its Fab mass in the mass analysis. The limitation of this approach is that the real structural and functional uniqueness of each clone, originating from an individual sequence for specific antigen binding, is not resolved. Similarly, related clones originating from the same ancestor would not be recognized as such, since we detect what is different and not what is the same. Ideally, we would also use mass spectrometry for the direct analysis of the full sequence of each clone detected in our LC-MS approach, even while present in the background of hundreds of other Ig clones. We feel that with further advances in top-, middle- and bottom-up proteomics this may become feasible soon (32–34). As proof-of-concept, we already demonstrated that a highly abundant IgG1 clone present in plasma could be fully sequenced, although this was still quite an arduous task (14). Improvements in middle- and top-down proteomics that specifically provide sequence information on the highly variable CDRs of the Fab fragments would be highly beneficial, as shown by others and us (35–37).

As little is known regarding the origin of human milk antibody repertoires, we can learn from other similar biological systems. For instance, by using both proteomics and genomics tools gut-derived repertoires have recently been shown to be related to blood Ig repertoires (29, 38–40). A similar approach could be used to help understand how human milk and blood repertoires are related, and further how all three systems, the gut, blood and human milk are interrelated. However, for this approach local plasma cells are used. The local population of the mammary gland is likely difficult to recognize, since there are also many antibody producing cells secreted into human milk (8). It is expected, though, that also several plasma cells are residing in the tissue, as we show that the human milk sIgA1 repertoire is highly stable for at least 16 weeks within an individual donor. Future efforts could therefore include direct clonal profiling of antibody repertoires as described here, hopefully soon supported by *de novo* sequencing by mass spectrometry, to further dissect clonal relatedness between tissues and body fluids.

In conclusion, the approach presented here can quantify the total IgA1 content in human milk. A unique advantage of this approach compared to for instance quantitative ELISA assays is that it allows for the monitoring of individual IgA1 clones, enabling us to observe how clonal profiles change over time, and to monitor at any given time which clones are contributing most to the total IgA1 concentration. In the future, the approach introduced here can be employed to monitor the humoral immune status in both human milk and plasma. Furthermore, it can be used to provide information on the personalized response towards an infection and/or therapeutic treatment through changes in the IgA1 clones being presented in a respective biofluid.

## Data Availability Statement 

The mass spectrometry data have been deposited to the MassIVE repository (https://massive.ucsd.edu/ProteoSAFe/static/massive.jsp) with the dataset identifier MSV000088188.

## Ethics Statement

Written informed consent was obtained prior to sample collection. All samples used were anonymized and donated to Danone Nutricia Research in accordance with the Helsinki Declaration II.

## Author Contributions

AB, KD and AH conceived the ideas and designed jointly the experiments. AB, KD, MH, and DR performed all experiments. MH performed a major part of the data analysis. All authors did write the manuscript together. AH provided resources and funding for the project. All authors contributed to the article and approved the submitted version.

## Funding

We acknowledge support from the Netherlands Organization for Scientific Research (NWO) funding the large-scale proteomics facility Proteins@Work (project 184.032.201) embedded in the Netherlands Proteomics Centre, Gravitation Subgrant 00022 from the Institute for Chemical Immunology (AB, DR, and AH), and the Spinoza award SPI.2017.028 to AH. Additional support for this research was provided by Danone Nutricia Research.

## Conflict of Interest

KD was enrolled as PhD student at Utrecht University during this study and received financial support from Danone Nutricia Research. The authors declare that the research was conducted in the absence of any commercial or financial relationships that could be constructed as a potential conflict of interest.

## Publisher’s Note

All claims expressed in this article are solely those of the authors and do not necessarily represent those of their affiliated organizations, or those of the publisher, the editors and the reviewers. Any product that may be evaluated in this article, or claim that may be made by its manufacturer, is not guaranteed or endorsed by the publisher.
