# Supplementary material for: A Direct MS-Based Approach to Profile Human Milk Secretory Immunoglobulin A (IgA1) Reveals Donor-Specific Clonal Repertoires With High Longitudinal Stability
Source: Front Immunol. 2021 Dec 6;12:789748. doi: 10.3389/fimmu.2021.789748 (PMC8685336; doi:10.3389/fimmu.2021.789748)
Supplement: Supplementary file 1 [file DataSheet_1.pdf]

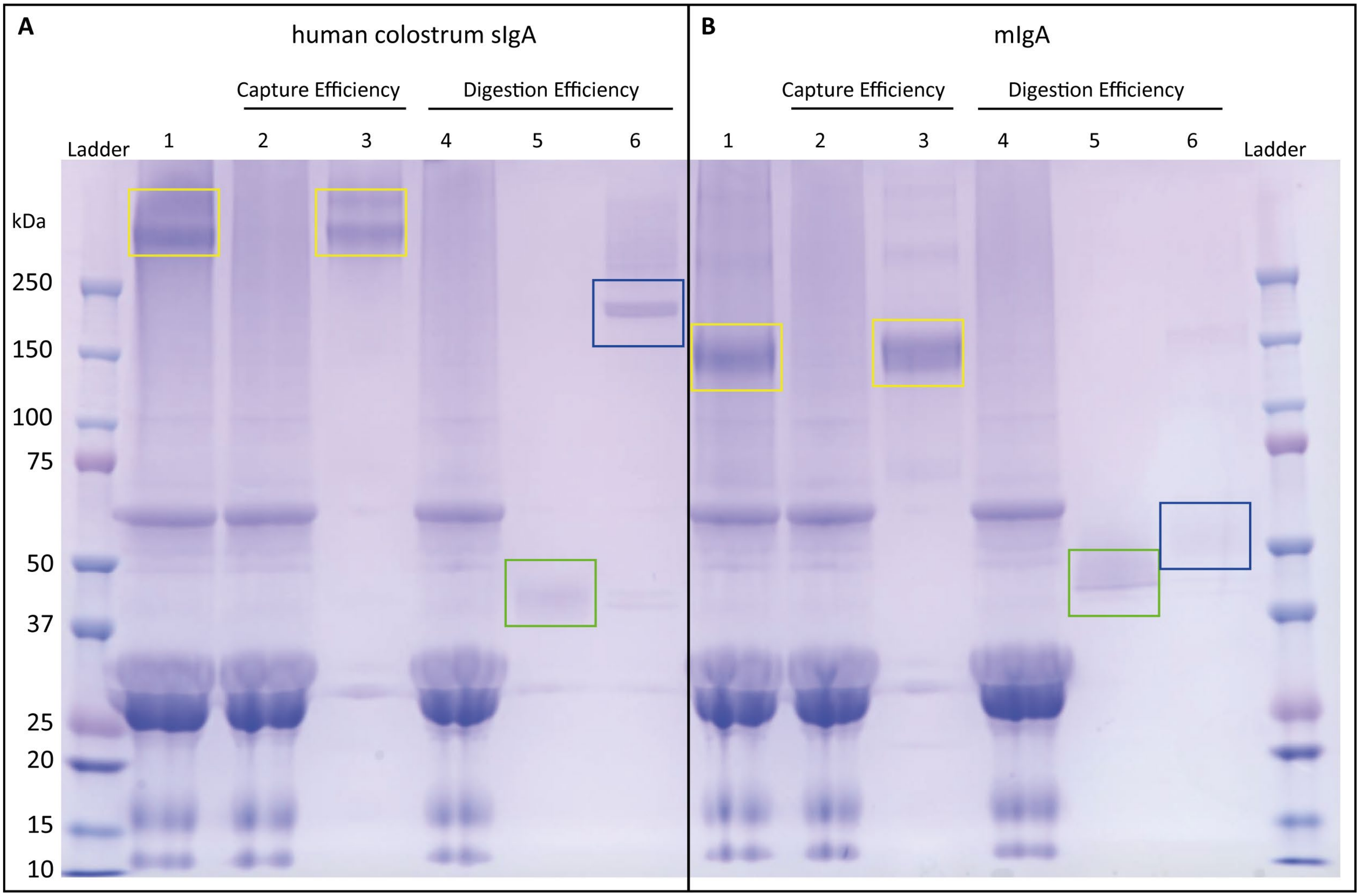

Figure S1 | Gel-based assessment of the IgA capturing and OgpA cleavage efficiency. SDS-PAGE gel of two different IgA standards A) human colostrum sIgA standard and B) monoclonal mIgA1. Both these standards were made at equivalent starting concentrations of 40ug in 1% milk powder background. Subsequently IgA was captured and digested by OgpA. On gel, samples were ran as 8 ug of protein each. From left to right: molecular weight ladder, 1) starting material, 2) flowthrough from the capturing, 3) eluant of the capture, 4) flowthrough from capturing, 5) Fab from digested IgA, and 6) eluant after digestion containing Fc. The expected starting material mass of IgA is indicated by the yellow boxes. The expected digested masses of the Fab and Fc are indicated in the green and blue boxes, respectively. Above the blue box in lane A6, the smear most likely indicates undigested sIgA2, only present in the human colostrum sIgA standard. The IgA masses of the two standards (lanes A1, A3, B1 and B3) are different as to the human colostrum sIgA is heavier due to additional J-chain and SC, which are not present in the mIgA1 standard. This also results in a heavier Fc for the human colostrum sIgA1 in lane A6. Both standards have Fab masses (lanes A5 and B5) at the expected 50 kDa mark. Additional bands on the gel are indicative of the milk proteins in the milk powder, namely: BSA at 60 kDa, casein fraction at 30 and 25 kDa, β-lactoglobulin at 16 kDa and α-lactoglobulin at 12 kDa, as confirmed by LC-MS based identification. In lanes A6 and B6 two bands are present at masses of ~40 kDa, these are the enzymes used for digestion of the Fab.

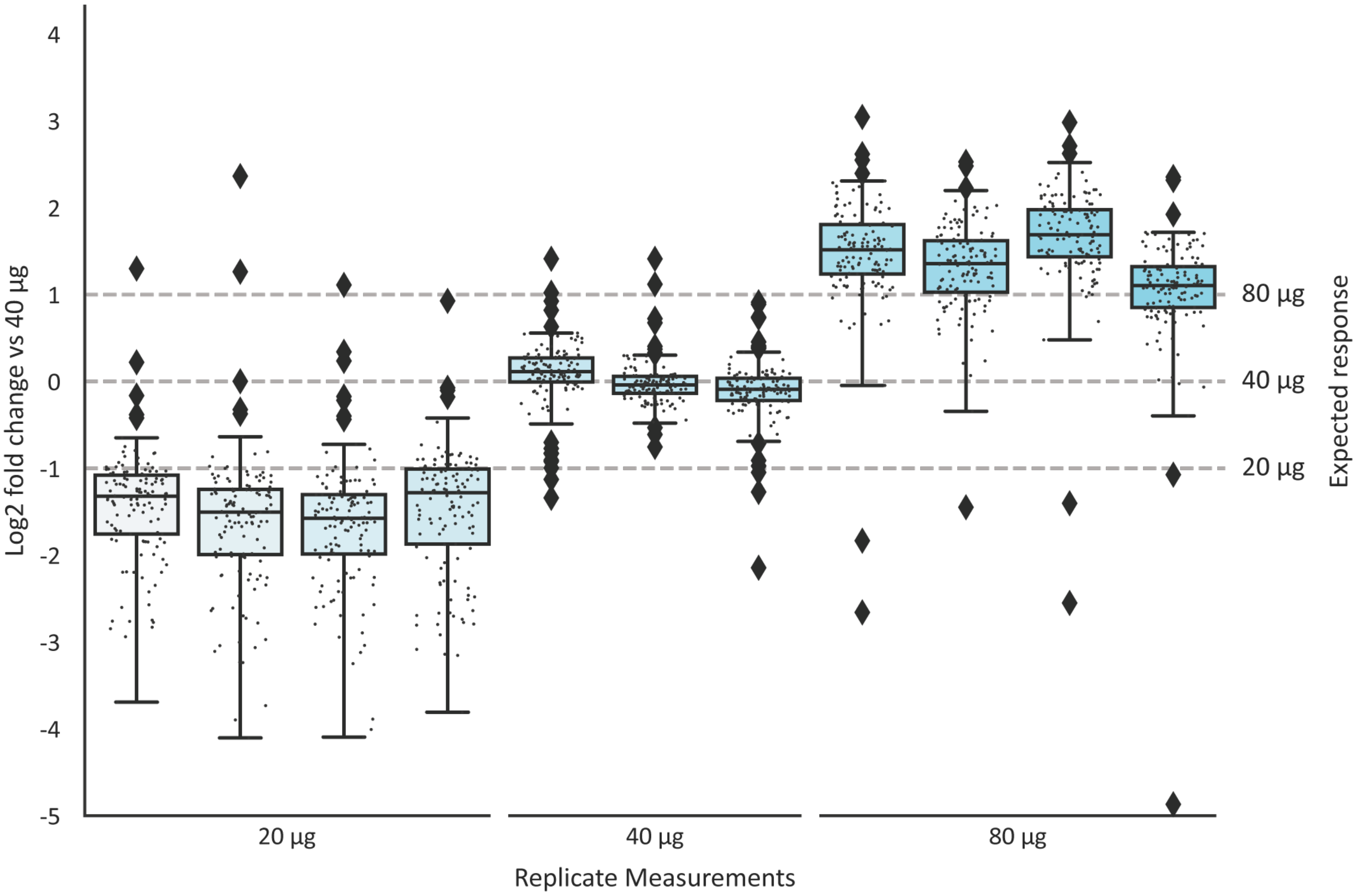

Figure S2 | Accuracy and reproducibility of quantification of individual sIgA clones.
All 128 clones that were detected in all replicates from a human milk colostrum sIgA standard were quantified relative to a monoclonal mIgA1 of known quantity. The detected quantities of each clone in all samples were compared to the levels in one sample with 40 µg sIgA, and the fold-change was plotted. Replicates of the same injection amount (40 µg) showed no significant fold-change. Clone intensities in the 20 µg replicates are detected at half the abundance (-1 $\log_2$ fold change), and clone intensity in the 80 µg replicates were detected at double the abundance. Boxplots indicate the median, and 25th and 75th percentile, whiskers range to 1.5 times IQR. All values inside this range are shown as black dots. Outliers (> 1.5 times IQR) are indicated as black diamonds.

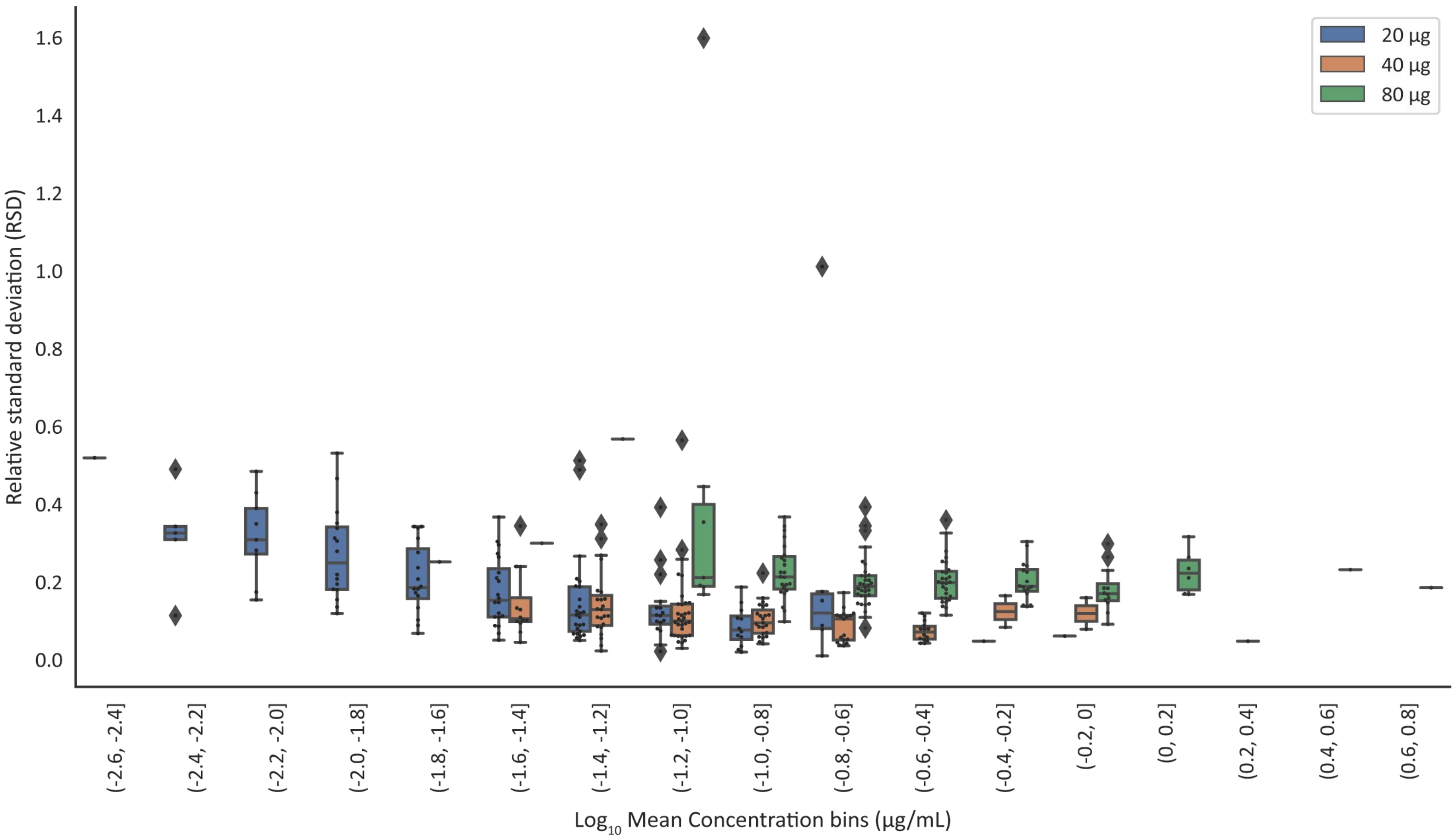

Figure S3 | Relative standard deviation of clone quantification.
Relative standard deviation (RSD) observed in the sIgA1 repertoire replicate analyses of a human milk colostrum sIgA standard. Boxplots indicate the median, 25th and 75th percentile, the whiskers range 1.5 times the inter-quartile range (IQR). Values outside this range are outliers, marked as black diamonds.

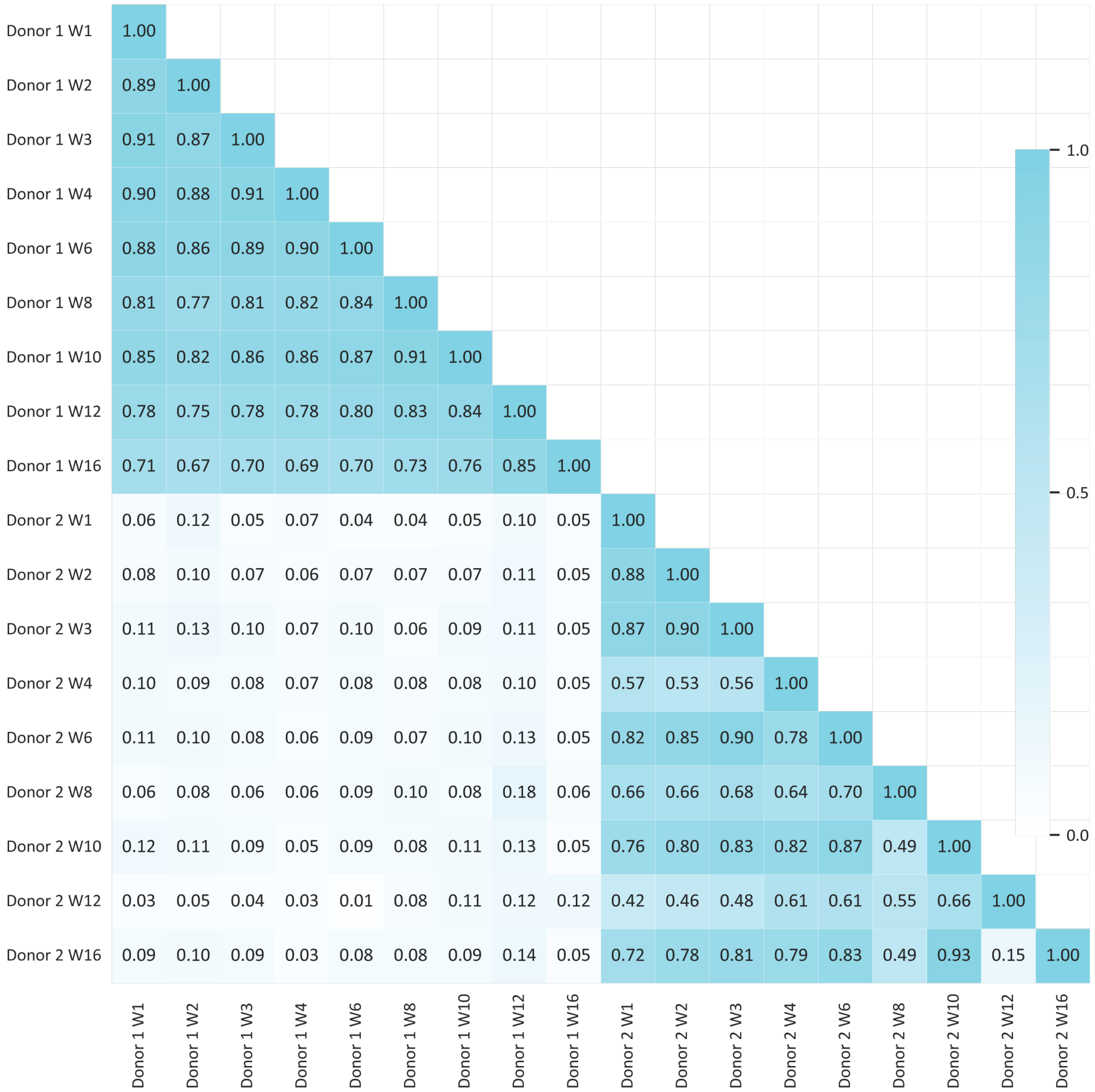

Figure S4 | Observed overlap in clonal sIgA1 repertoires.

Observed overlap within and in between donors. The persistence of repertoires is given as a percentage of the total sIgA1 clone abundance. Each small square depicts a percentage, as indicated by the color bar and the value inside the square, of overlap between the samples. This figure represents a value annotated copy of **Figure 4B**.

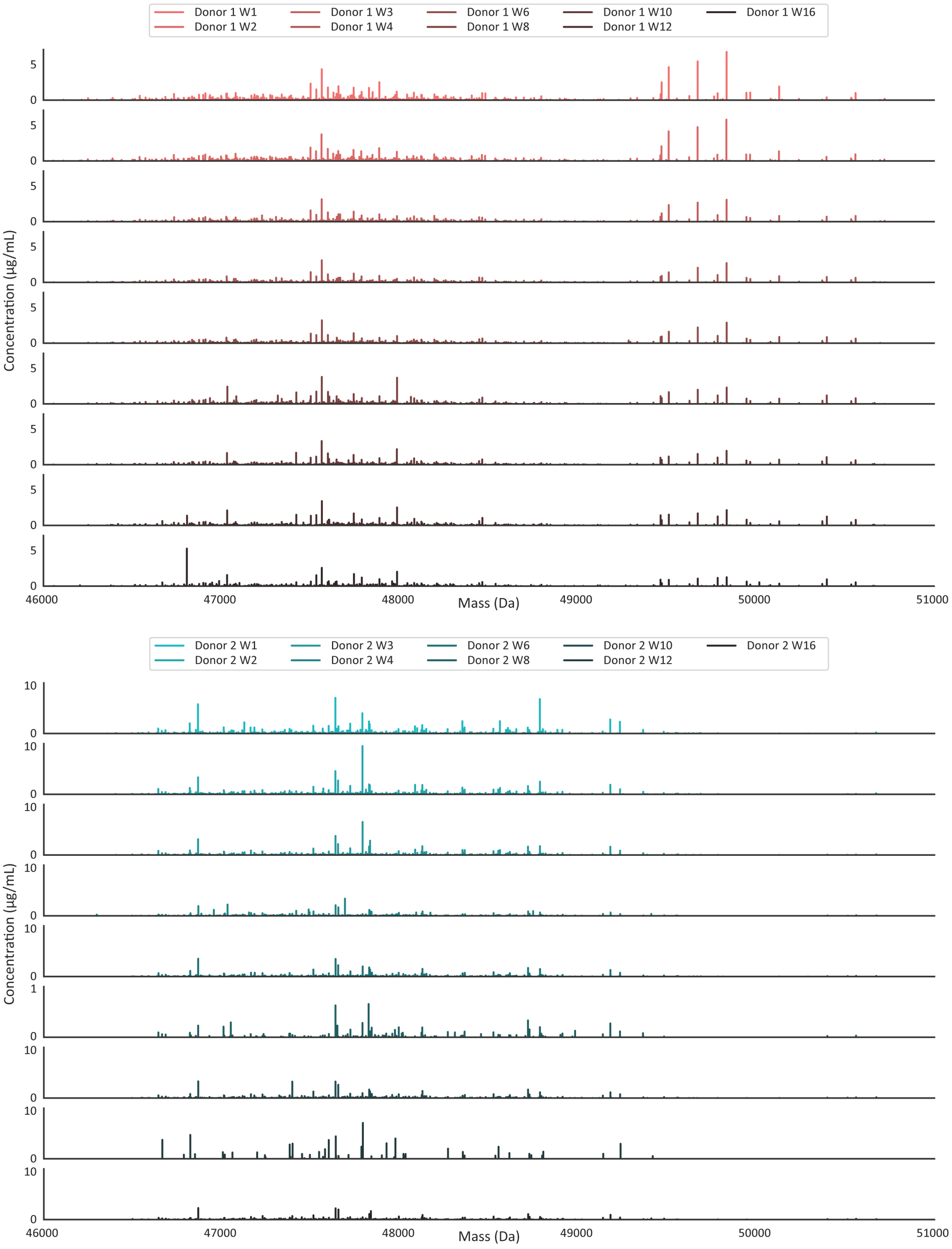

Figure S5 | Mass profiles of Fab repertoires derived from human milk sIgA1 in samples from both donors at all sampled timepoints. Each peak represents a unique Fab at its detected mass and concentration in human milk. Clearly within a donor these sIgA1 Fab profiles look very much alike, but between donors they are strikingly dissimilar. All profiles were compared with each other and used to prepare the correlation matrix shown in **Figure 4 and Figure S4**.

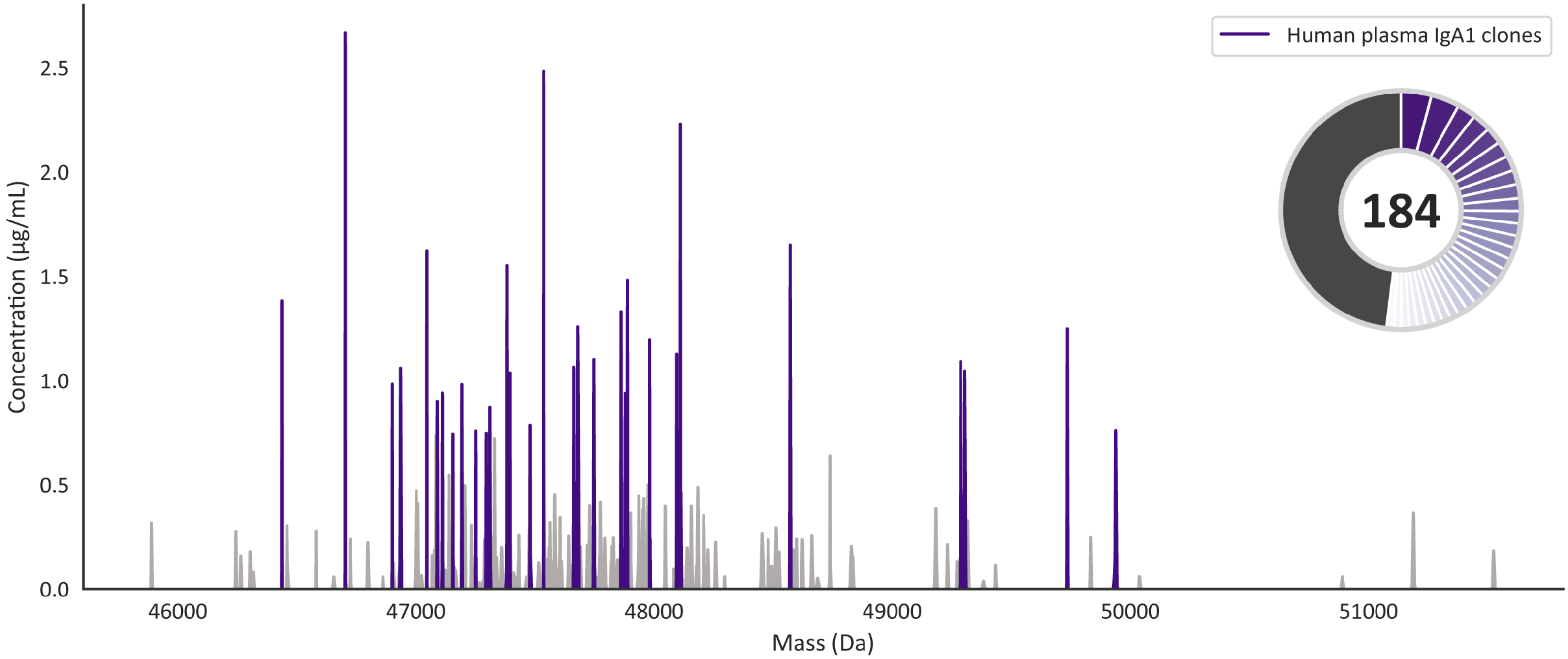

Figure S6 | The IgA1 repertoire profiling approach performs equally well in serum. Proof-of-concept data, showing the mass profile of Fabs derived from human plasma IgA1 from a single healthy donor. Each peak represents a unique Fab at its detected mass and concentration in human serum. The top 30 most abundant clones are colored purple, while the remainder are colored grey. The number in the pie inset shows the total number of identified Fab clones. The contribution to the total concentration of the top 30 most abundant clones is depicted in order of abundance by the pie slices, the remainder of the clones are depicted as the black pie slice. This reveals that of the total concentration of identified IgA1 Fab clones, the top 30 most abundant contribute about 50% to the total repertoire present in the plasma.
