# Supplementary material for: A Direct MS-Based Approach to Profile Human Milk Secretory Immunoglobulin A (IgA1) Reveals Donor-Specific Clonal Repertoires With High Longitudinal Stability
Source: Front Immunol. 2021 Dec 6;12:789748. doi: 10.3389/fimmu.2021.789748 (PMC8685336; doi:10.3389/fimmu.2021.789748)
Supplement: Supplementary file 2 [file Table_1.docx]

**Supplemental Table S1** Monoclonal IgA1 (7D8) Fab sequence

| ***Fd*** | ***LC*** | ***Fab mass (avg)*** |
| --- | --- | --- |
| EVQLVESGGGLVQPDRSLRLSCAASGFTFHDYAMHWVRQAPGKGLEWVSTISWNSGTIGYADSVKGRFTISRDNAKNSLYLQMNSLRAEDTALYYCAKDIQYGNYYYGMDVWGQGTTVTVSSASPTSPKVFPLSLCSTQPDGNVVIACLVQGFFPQEPLSVTWSESGQGVTARNFPPSQDASGDLYTTSSQLTLPATQCLAGKSVTCHVKHYTNPSQDVTVPCPVPS | EIVLTQSPATLSLSPGERATLSCRASQSVSSYLAWYQQKPGQAPRLLIYDASNRATGIPARFSGSGSGTDFTLTISSLEPEDFAVYYCQQRSNWPITFGQGTRLEIKRTVAAPSVFIFPPSDEQLKSGTASVVCLLNNFYPREAKVQWKVDNALQSGNSQESVTEQDSKDSTYSLSSTLTLSKADYEKHKVYACEVTHQGLSSPVTKSFNRGEC | 47957.41 |

**Supplemental Table S2** Serial dilution for quantification of sIgA and mIgA standards^*^

| **mAb** | **Human colostrum sIgA** |
| --- | --- |
| 200 ng | 20 µg |
| 200 ng | 40 µg |
| 200 ng | 80 µg |

^*^All samples were made in a 1% milk powder background in PBS.
